# Supplementary material for: Affinity for DNA Contributes to NLS Independent Nuclear Localization of MeCP2
Source: Cell Rep. 2018 Aug 28;24(9):2213–20. doi: 10.1016/j.celrep.2018.07.099 (PMC6130050; doi:10.1016/j.celrep.2018.07.099)
Supplement: Document S1. Figures S1 and S2 [file mmc1.pdf]

Cell Reports, Volume *24*

Supplemental Information

Affinity for DNA Contributes to NLS Independent Nuclear Localization of MeCP2

Matthew J. Lyst, Robert Ekiert, Jacky Guy, Jim Selfridge, Martha V. Koerner, Cara Merusi, Dina De Sousa, and Adrian Bird

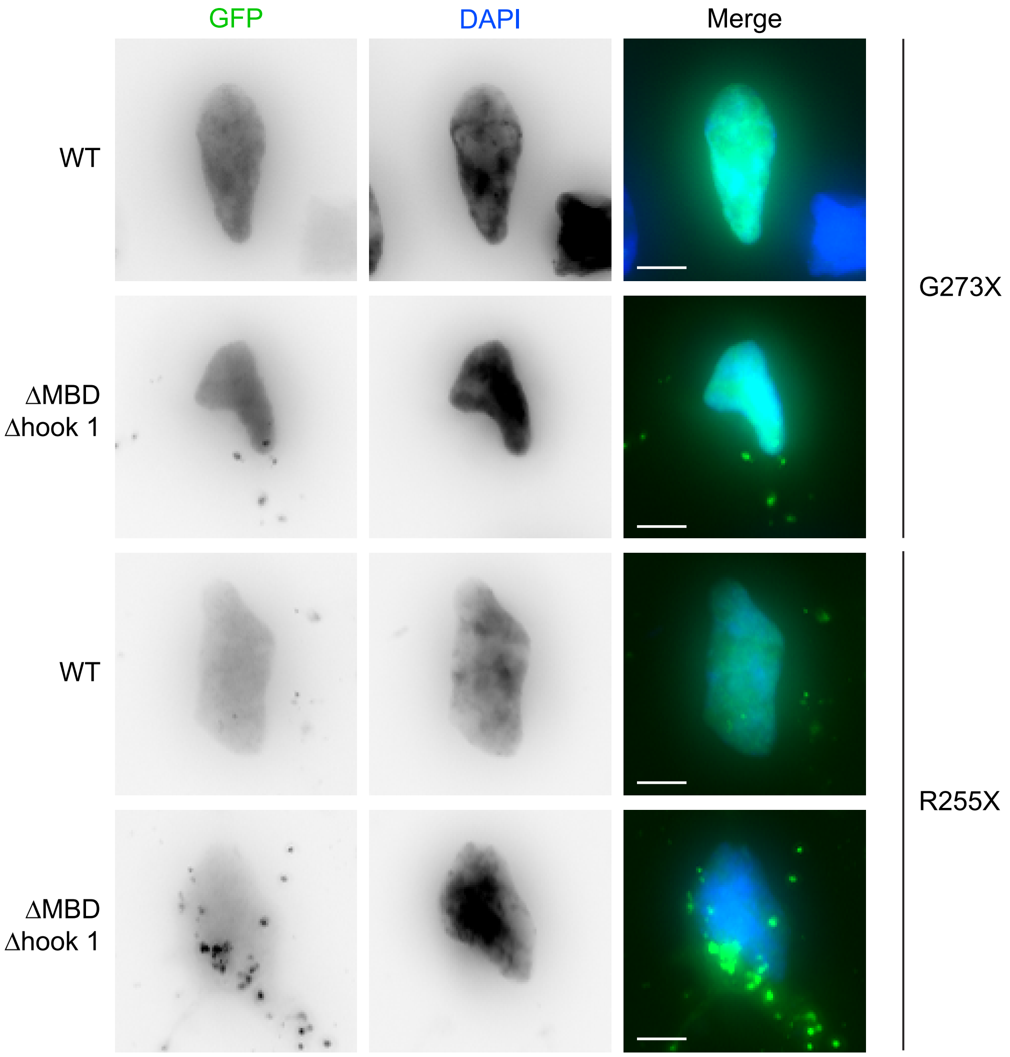

Figure S1. Redundancy between DNA binding and the NLS for nuclear localization of MeCP2 expressed in the human neuronal LUHMES cell line. Related to Figure 2.

Lentivirally expressed EGFP-MeCP2 with an intact NLS (G273X truncation) is nuclear and co-localizes with DAPI stain regardless of whether DNA binding is wild-type (WT) (top panels) or disturbed (ΔMBDΔhook 1) (second panels). When the NLS is removed (R255X truncation) EGFP-MeCP2 remains nuclear when the DNA binding domains are intact (WT) (third panels), but becomes largely cytoplasmic when the DNA binding domains are mutated (ΔMBDΔhook 1) (bottom panels). The scale bar represents 5 µm.

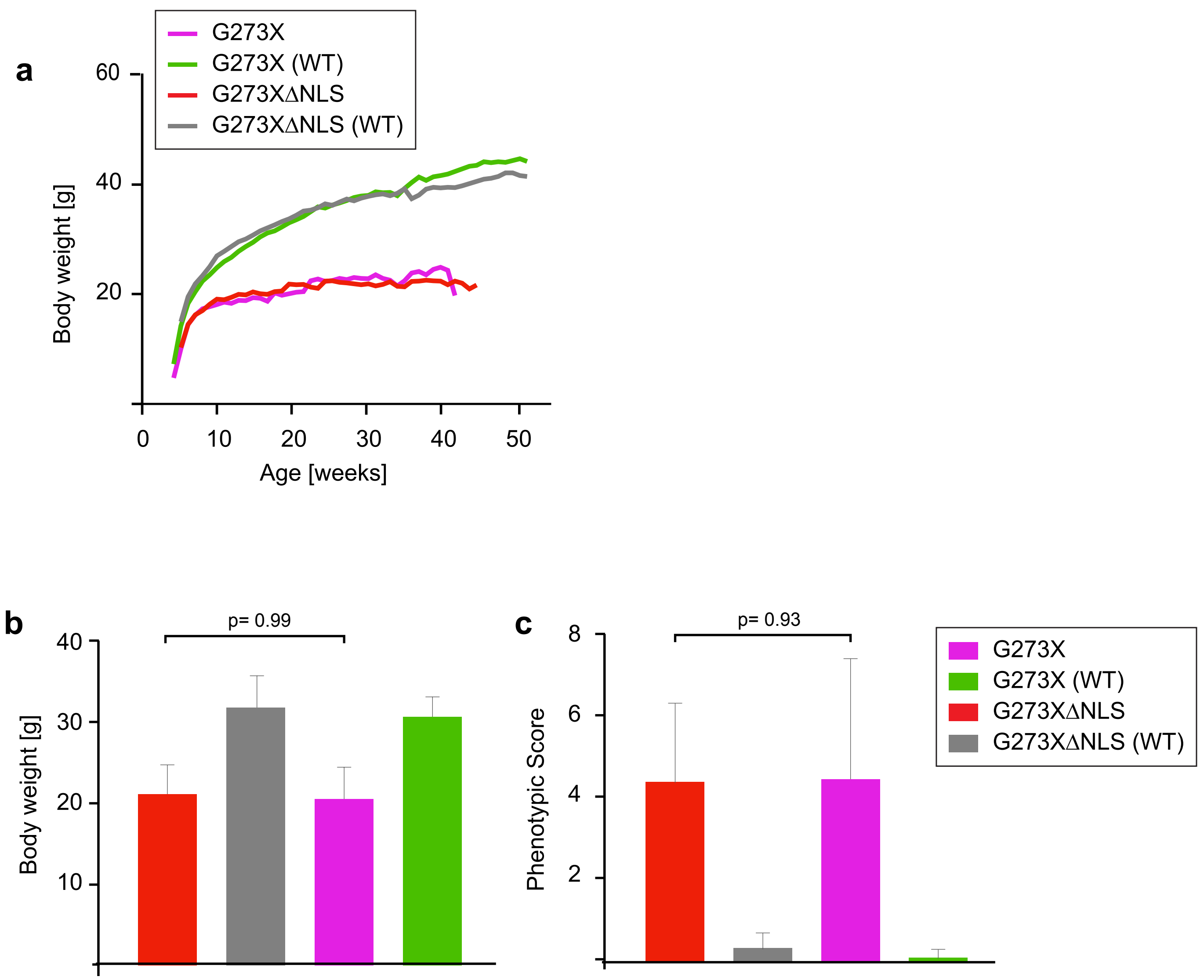

Figure S2. Body weights and phenotypic scores of MeCP2^{G273X}-EGFP and MeCP2$^{G273X\Delta NLS}$-EGFP mice. Related to Figure 4.

(a) Average body weight of surviving animals is plotted against age for each group. Data for each group plotted up to the last point when there were at least three surviving animals. **(b)** Body weights of each group of animals at 15-weeks – the last time point to which all mice survived. Data are presented as means ± standard deviation. No significant difference was observed between MeCP2^{G273X}-EGFP and MeCP2$^{G273X\Delta NLS}$-EGFP mice (p=0.99; Kolmogorov-Smirnov test). **(c)** Phenotypic scores of each group of animals at 15-weeks – the last time point to which all of the mice survived. Data are presented as means ± standard deviation. No significant difference was observed between MeCP2^{G273X}-EGFP and MeCP2$^{G273X\Delta NLS}$-EGFP mice (p=0.93; Kolmogorov-Smirnov test).
