# Supplementary material for: Affinity for DNA Contributes to NLS Independent Nuclear Localization of MeCP2
Source: Cell Rep. 2018 Aug 28;24(9):2213–20. doi: 10.1016/j.celrep.2018.07.099 (PMC6130050; doi:10.1016/j.celrep.2018.07.099)
Supplement: Document S2. Article plus Supplemental Information [file mmc2.pdf]

Cell Reports

Report

Affinity for DNA Contributes to NLS Independent Nuclear Localization of MeCP2

Graphical Abstract

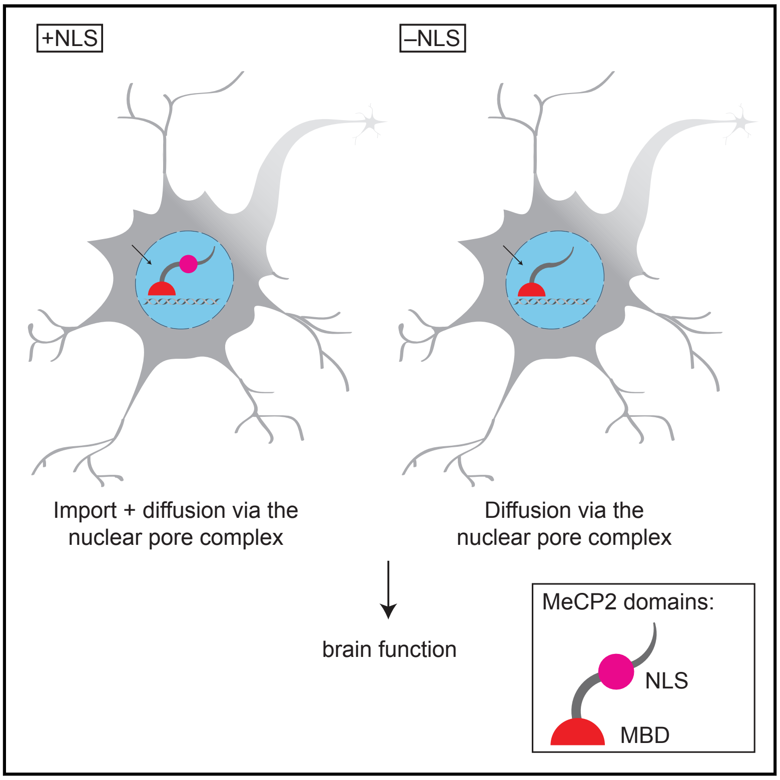

Highlights

- Nuclear localization of MeCP2 does not require its NLS
- DNA binding by MeCP2 contributes to its NLS-independent nuclear localization
- MeCP2 NLS mutation does not affect pathology in a mouse model of Rett syndrome

Authors

Matthew J. Lyst, Robert Ekiert, Jacky Guy, ..., Cara Merusi, Dina De Sousa, Adrian Bird

Correspondence

a.bird@ed.ac.uk

In Brief

Lyst et al. report that nuclear localization of MeCP2 does not require its nuclear localization signal. Instead, this protein is efficiently partitioned in the nucleus via its affinity for DNA. Consistent with this finding, mutating the MeCP2 NLS does not affect pathology in a mouse model of Rett syndrome.

Lyst et al., 2018, Cell Reports *24*, 2213–2220
August 28, 2018 © 2018 The Authors.
https://doi.org/10.1016/j.celrep.2018.07.099

CellPress

Cell Reports
Report

OPEN ACCESS
CellPress

Affinity for DNA Contributes to NLS Independent Nuclear Localization of MeCP2

Matthew J. Lyst,[1] Robert Ekiert,[2] Jacky Guy,[1] Jim Selfridge,[1] Martha V. Koerner,[1] Cara Merusi,[1] Dina De Sousa,[1] and Adrian Bird[1,3,*]
[1]Wellcome Centre for Cell Biology, University of Edinburgh, Michael Swann Building, Max Born Crescent, The King's Buildings, Edinburgh EH9 3BF, UK
[2]Department of Molecular Biophysics, Faculty of Biochemistry, Biophysics and Biotechnology, Jagiellonian University, 30-387 Kraków, Poland
[3]Lead Contact
*Correspondence: a.bird@ed.ac.uk
https://doi.org/10.1016/j.celrep.2018.07.099

SUMMARY

MeCP2 is a nuclear protein that is mutated in the severe neurological disorder Rett syndrome (RTT). The ability to target β-galactosidase to the nucleus was previously used to identify a conserved nuclear localization signal (NLS) in MeCP2 that interacts with the nuclear import factors KPNA3 and KPNA4. Here, we report that nuclear localization of MeCP2 does not depend on its NLS. Instead, our data reveal that an intact methyl-CpG binding domain (MBD) is sufficient for nuclear localization, suggesting that MeCP2 can be retained in the nucleus by its affinity for DNA. Consistent with these findings, we demonstrate that disease progression in a mouse model of RTT is unaffected by an inactivating mutation in the NLS of MeCP2. Taken together, our work reveals an unexpected redundancy between functional domains of MeCP2 in targeting this protein to the nucleus, potentially explaining why NLS-inactivating mutations are rarely associated with disease.

INTRODUCTION

Eukaryotic cells are defined by having their genetic material contained within a membrane-bound organelle known as the nucleus. The nuclear pore complex (NPC) is a large protein channel embedded in the nuclear membrane that mediates transport of macromolecules between the nucleus and the cytoplasm. Small proteins are able to diffuse passively through the NPC, whereas larger factors require a nuclear localization signal (NLS)—a short motif that binds to proteins mediating active transport through the NPC (Sorokin et al., 2007). One such factor thought to require NLS-dependent transport into the nucleus is the chromatin-associated protein, MeCP2.

MeCP2 has been subjected to intense study since the discovery that mutations in this factor cause Rett syndrome (RTT)—a severe neurological disorder affecting approximately 1 in 10,000 girls (Amir et al., 1999). The original identification of MeCP2 was based on the affinity of its methyl-CpG binding domain (MBD) for DNA containing methylated CpG dinucleotides (Lewis et al., 1992; Nan et al., 1993). RTT-causing missense mutations cluster within the MBD and impair the ability of MeCP2 to interact with chromatin (Baubec et al., 2013; Christodoulou et al., 2003; Goffin et al., 2011), and so MBD-mediated binding to DNA is likely to be essential for MeCP2 function. Analysis of brain regions from MeCP2-deficient mice revealed a slight relative increase in the expression levels of many genes with the greatest number of methylated cytosines, suggesting that MeCP2 functions as a weak but global transcriptional repressor (Gabel et al., 2015; Kinde et al., 2016; Lagger et al., 2017). Consistent with this view, a second cluster of RTT-causing mutations in MeCP2 destroys its interaction with the NCOR1/NCOR2 co-repressor complex (Kruusvee et al., 2017; Lyst et al., 2013). Alternative models of MeCP2 molecular function have also been proposed, including roles in transcriptional activation (Chahrour et al., 2008; Li et al., 2013), alternative splicing (Young et al., 2005), microRNA processing (Cheng et al., 2014), and chromatin compaction (Baker et al., 2013). Although, the precise connection between these putative MeCP2 functions and RTT pathology has yet to be firmly established, each model nevertheless requires the presence of MeCP2 in the nucleus (Lyst and Bird, 2015).

In this study, we reveal a redundancy in the mechanism by which MeCP2 is localized to the nucleus. We report that nuclear localization of MeCP2 is not exclusively dependent on its canonical NLS. Rather, we find that either an intact NLS or chromatin binding is sufficient for nuclear retention. Consistent with NLS-independent nuclear localization of MeCP2, we find that disease progression in a mouse model of Rett syndrome is not impacted by a mutation that abolishes binding of the MeCP2 NLS to the nuclear import factors Kpna3 and Kpna4. Taken together, our results reveal a functional redundancy between DNA binding and the NLS in targeting MeCP2 to the nucleus. This may rationalize the absence of pathogenic mutations in the NLS of MeCP2 and may also be considered as an explanation for the scarcity of disease-causing mutations in the NLS motifs of other factors (McLane and Corbett, 2009).

RESULTS

The NLS of MeCP2 Interacts with KPNA3 and KPNA4

Previously, we affinity purified MeCP2 from the brains of *Mecp2-EGFP* knockin mice and identified the nuclear import factors

This is an open access article under the CC BY license (http://creativecommons.org/licenses/by/4.0/).

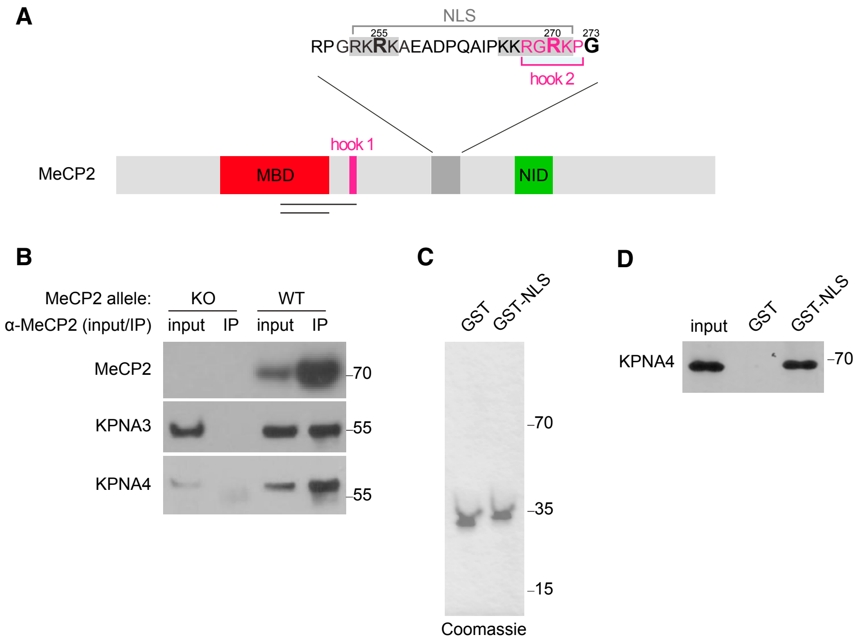

Figure 1. The NLS of MeCP2 Interacts with KPNA3 and KPNA4

(A) Schematic of the primary structure of MeCP2 showing the positions of the methyl-CpG binding domain (MBD), the first AT-hook (hook 1), the nuclear localization signal (NLS), and the NCOR1/NCOR2 interaction domain (NID). The expanded view of the NLS depicts the two basic patches of this motif (gray), the overlapping AT-hook 2 (hook 2) (pink), and the positions of the RTT-causing truncation mutations R255X, R270X, and G273X (bold). Horizontal black lines indicate the extent of the deletions used in the MBD and in AT-hook 1.

(B) Immunoprecipitation (IP) of MeCP2, Kpna3, and Kpna4 from extracts of wild-type (WT) mouse brain using an antibody against MeCP2. IP from *Mecp2* knockout (KO) brain is a negative control.

(C) Coomassie stain of purified GST-MeCP2^{NLS} (GST-NLS) and GST alone.

(D) Western blotting reveals *in vitro*-translated 3×FLAG-tagged Kpna4 is bound by GST-NLS, but not by GST alone.

Kpna3 and Kpna4 as binding partners by mass spectrometry (Lyst et al., 2013). Subsequently, others mapped the KPNA3/KPNA4 binding site on MeCP2 to its previously identified NLS (Baker et al., 2015; Nan et al., 1996). This bipartite NLS is located between the MBD and the NCOR1/NCOR2 interaction domain (NID) of MeCP2 (Figure 1A).

To validate the mass spectrometry data and to ensure that these interactions are not artifacts of the EGFP tag, we performed immunoprecipitation from wild-type mouse brain nuclear extracts using an antibody against MeCP2 and revealed bound Kpna3/Kpna4 by western blotting. These proteins were absent in control immunoprecipitations performed using extracts from *Mecp2*-null mice, demonstrating the specificity of this assay. MeCP2 binding to Kpna3/Kpna4 was still readily detectable even using stringent washes with 300 mM sodium chloride, suggesting that this interaction is robust (Figure 1B). Next, we tested whether the MeCP2 NLS is sufficient to bind directly to the nuclear import machinery. To this end, we bacterially expressed and purified the MeCP2 NLS as a glutathione S-transferase (GST) fusion and immobilized this protein on glutathione Sepharose (Figure 1C). This protein was able to pull down recombinant 3×FLAG-tagged Kpna4 produced by *in vitro* translation in rabbit reticulocyte lysate. GST alone, on the other hand, showed no binding to Kpna4 in this assay (Figure 1D). Together with previous observations, our data strongly suggest that the MeCP2 NLS interacts directly with the nuclear import machinery *in vivo*.

The MBD of MeCP2 Is Sufficient for Nuclear Localization

Previous studies have shown that MeCP2 fused to EGFP localizes primarily to the nucleus, even in the presence of truncation mutations that disturb the originally defined NLS (Baker et al., 2013, 2015; Kumar et al., 2008; Schmiedeberg et al., 2009). Possible explanations for these observations could be residual activity of the canonical NLS or the presence of an unidentified NLS in MeCP2, which is not affected by these truncation mutations. An alternative hypothesis—not mutually exclusive with these models—is that MeCP2 might pass through nuclear pore complexes in a NLS-independent manner and then be retained in the nucleus due to its affinity for DNA.

To investigate these scenarios, we introduced a deletion mutation into the MBD—the primary DNA binding domain of MeCP2. This DNA binding mutant was then combined with truncation mutations, which either preserve or disrupt the NLS. The resulting proteins were then expressed in NIH 3T3 cells as fusions with EGFP. MeCP2 G273X, which has a wild-type MBD and preserves the NLS, localized correctly in the nucleus concentrating at DAPI dense heterochromatic foci (Figure 2A, upper left). Disrupting the NLS with the R255X mutation had only a minor effect on this localization, with the protein remaining overwhelmingly nuclear and concentrated in heterochromatic foci (Figure 2A, upper right). Nevertheless, analysis of numerous cells expressing this mutant allowed for the detection of a statistically significant increase in the amount of cytoplasmic EGFP-MeCP2 (Figure 2B). Deletion of the MBD resulted in the release of MeCP2 G273X from heterochromatin, but the protein remained predominantly nuclear (Figure 2A, middle left). When, however, the MBD was mutated in the context of the R255X truncation, which removes the NLS, MeCP2 was readily detectable in the cytoplasm (Figure 2A, middle right), indicating that affinity for DNA might contribute to nuclear localization of MeCP2.

DNA Binding Correlates with NLS Independent Nuclear Retention of MeCP2

To probe further the relationship between nuclear retention and affinity for DNA, we extended the deletion mutant in the MBD to include the adjacent DNA binding motif, AT-hook 1 (Figure 1A).

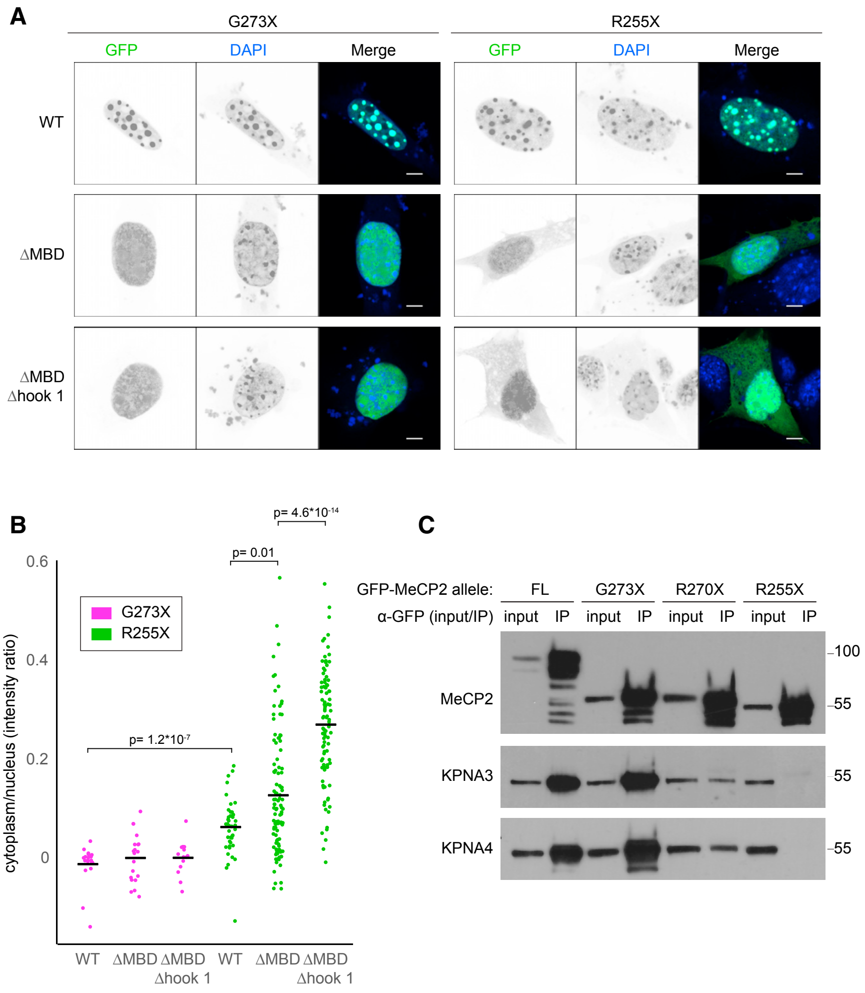

Figure 2. Importin Binding Is Not Required for MeCP2 Retention in the Nucleus

(A) NIH 3T3 cells expressing mutated forms of MeCP2 as EGFP fusion proteins and counter-stained with DAPI. The NLS of MeCP2 is intact (G273X) or truncated (R255X), and the DNA binding domains are WT, impaired (ΔMBD), or severely impaired (ΔMBDΔhook 1). The scale bars represent 5 μm.

(B) Quantification of ratio of cytoplasmic to nuclear signal intensity of EGFP-MeCP2 in above panels. Data points represent individual cells. Horizontal bars represent means. Statistical significance was calculated using the Wilcoxon test.

(C) IP using an antibody against EGFP of EGFP-MeCP2, KPNA3, and KPNA4 from extracts of HeLa cells expressing full-length (FL), G273X, R270X, and R255X forms of EGFP-MeCP2.

See also Figure S1.

AT-hook 1 is a sequence-specific DNA binding motif that contributes to chromatin binding by MeCP2 (Lyst et al., 2016). When this deletion was combined with G273X, the NLS of MeCP2 was nevertheless able to direct efficient nuclear localization (Figure 2A, bottom left). However, in combination with R255X, this mutation led to increased cytoplasmic accumulation of MeCP2 (Figure 2A, bottom right). Notably, in the absence of the NLS, simultaneous inactivation of the MBD and AT-hook 1 leads to a greater degree of cytoplasmic localization than that caused by abrogation of the MBD alone (Figure 2B). Therefore, the data support the conclusion that only inactivation of both the NLS and the ability to bind to DNA results in substantial cytoplasmic accumulation of MeCP2 and that the degree of nuclear retention correlates well with the ability of NLS-mutated versions of MeCP2 to bind to DNA (Figure 2B).

A Role for DNA Binding in Nuclear Localization of MeCP2 in Human Neurons

Given that RTT is a neurological disorder, we next asked whether this redundancy between the NLS and the DNA binding domains of MeCP2 could be observed in a more physiologically relevant neuronal culture system. In particular, we employed the human LUHMES cell line, which can be efficiently differentiated into mature post-mitotic dopaminergic neurons *in vitro* (Scholz et al., 2011). Using a lentiviral expression system to produce EGFP-MeCP2 in these cells, we observed that both MeCP2 G273X and MeCP2 R255X as well as MeCP2 G273XΔMBDΔAT-hook 1 were all found in the nucleus as assessed by co-localization with DAPI-stained DNA (Figure S1, upper three panels). MeCP2 R255XΔMBDΔAT-hook 1, on the other hand, the only mutant protein with the DNA binding domains of MeCP2 inactivated together with its NLS, showed minimal co-localization with DAPI-stain nuclear DNA (Figure S1, bottom panels). Strikingly, the majority of the EGFP signal was found in aggregates outside of the nucleus, suggesting that, in this system, the DNA binding domains of MeCP2 play a key role in directing NLS-independent nuclear localization of MeCP2. We therefore conclude that the redundancy between the NLS and the DNA binding domains of MeCP2, which we first observed in mouse fibroblasts, is a general phenomenon that also holds true in human neurons.

MeCP2 R270X Retains a Residual Interaction with KPNA3/KPNA4

A previous study reported that the R270X mutation in MeCP2 abolishes its binding to KPNA3 but preserves a residual interaction with KPNA4 (Baker et al., 2015). The authors proposed that this weak binding to KPNA4 was likely to explain the nuclear localization of MeCP2 R270X. In our hands, as assessed by co-immunoprecipitation with exogenously expressed EGFP-MeCP2 in HeLa cells, both KPNA3 and KPNA4 show strongly reduced binding to the R270X mutation. However, both still retain a residual interaction compared with the more severe R255X truncation, which does not bind detectably to KPNA3 or

KPNA4 (Figure 2C). Given that an intact MBD is able to direct nuclear targeting of MeCP2 R255X, this weak binding of MeCP2 R270X to KPNA3/KPNA4 is unlikely to be necessary for the nuclear localization of this mutant protein. Overall, therefore, we conclude that MeCP2 is able to enter the nucleus in an importin-independent manner and that it can then be partitioned there due to binding to chromosomal DNA.

NLS Inactivation Does Not Influence MeCP2 Level or Localization in Mice

An earlier study found that the MeCP2 R270X mutation gives rise to more severe neurological deficits than the MeCP2 G273X mutation in mice. These phenotypic differences were attributed to the presence of an AT-hook in this region of the protein (AT-hook 2; Figure 1A) rather than any effects of the overlapping NLS, due to the nuclear localization of MeCP2 R270X (Baker et al., 2013). However, despite proper nuclear localization of MeCP2 R270X, loss of a non-canonical function of importin interaction contributing to the phenotypic deficits in these animals could not be excluded.

To assess the functional relevance of the MeCP2 NLS, we generated mutant mice designed to distinguish the effect of AT-hook 2 from that of importin binding. The first animal model carried a truncation mutation of MeCP2, where every amino acid from G273 is removed and replaced with a linker followed by EGFP. This essentially recapitulates an earlier model (Baker et al., 2013), except that the mutation is knocked into the endogenous locus rather than being expressed as a transgene. A second line was constructed similarly, except that four basic residues in the N-terminal part of the NLS were mutated to alanine. These residues do not overlap with AT-hook 2 (Figure 1A), and so this mutation allowed us to separate the phenotypic consequences of inactivating the NLS or this AT-hook (Figure 1A). We designated these two lines as *Mecp2*^{G273X}-*EGFP* and *Mecp2*$^{G273X\Delta NLS}$-*EGFP*.

We first checked the expression levels of MeCP2 in these mice. As assessed by western blotting, total MeCP2 was slightly increased above that in wild-type controls, closely mirroring the situation when full-length MeCP2 is tagged with EGFP (Figure 3A; Brown et al., 2016). To confirm that the MeCP2$^{G273X\Delta NLS}$-EGFP mutation abolished the interaction of MeCP2 with Kpna3/Kpna4, we performed immunoprecipitation using an antibody against EGFP followed by western blotting with antibodies against KPNA3 and KPNA4. Whereas Kpna3 and Kpna4 bind robustly to MeCP2^{G273X}-EGFP, no interaction could be detected with MeCP2$^{G273X\Delta NLS}$-EGFP, even though mild buffers containing only 150 mM sodium chloride were used (Figure 3B).

Next, we investigated the subcellular localization of MeCP2^{G273X}-EGFP and MeCP2$^{G273X\Delta NLS}$-EGFP. Imaging of EGFP in CA1 hippocampal neurons in brain sections from these mice revealed that both mutant proteins localize correctly to DAPI dense foci, demonstrating that the NLS is not necessary for proper nuclear localization of MeCP2 *in vivo* (Figure 3C). To confirm the exclusively nuclear localization of these proteins, we prepared whole-cell and cytoplasmic extracts from these brains. Western blotting revealed that, unlike the control protein, gamma-tubulin, MeCP2 was found only in the whole-cell extract and was completely absent from the cytoplasmic fractions (Figure 3D). From these observations, we conclude that, in the brain, MeCP2^{G273X}-EGFP remains strictly nuclear, even when the interaction of its NLS with Kpna3/Kpna4 is lost. The dispensability of the MeCP2 NLS that we have uncovered in tissue culture systems therefore appears to be also applicable in the most physiologically relevant tissue for this protein—the brain.

MeCP2 NLS Inactivation Does Not Influence the RTT-like Phenotype in Mice

As the *Mecp2*^{G273X}-*EGFP* and *Mecp2*$^{G273X\Delta NLS}$-*EGFP* mice express MeCP2 to similar levels, we used these animals to assess the phenotypic consequences of destroying the interaction of MeCP2 with Kpna3/Kpna4. Like other RTT models, both lines initially developed grossly normally before succumbing to various neurological deficits. In order to make an objective comparison of the phenotypic severity in these two lines, we monitored the survival times and body weights of cohorts of male mice hemizygous for the *Mecp2* gene. Consistent with the previous report on the G273X mutation, our *Mecp2*^{G273X}-*EGFP* line displayed a median survival time of 36 weeks (Baker et al., 2013). This is substantially longer than the median survival time of 8 weeks reported for *Mecp2*-null mice (Baker et al., 2013; Chen et al., 2001; Guy et al., 2001). Our *Mecp2*$^{G273X\Delta NLS}$-*EGFP* mice also survived longer than null animals with a median age at death of 41 weeks. This is comparable to survival of the *Mecp2*^{G273X}-*EGFP* animals, with no statistically significant difference detected between the two groups (Figure 4A). Also consistent with previous findings on *Mecp2*-null mice (Guy et al., 2001), we observed that animals from both lines were underweight when compared to wild-type controls (Figure S2A). However, in keeping with the similar survival curves of *Mecp2*^{G273X}-*EGFP* and *Mecp2*$^{G273X\Delta NLS}$-*EGFP* mice, we did not detect a significant difference between the body weights of these two lines (Figure S2B).

To further characterize the *Mecp2*^{G273X}-*EGFP* and *Mecp2*$^{G273X\Delta NLS}$-*EGFP* mice, we employed a phenotypic scoring system designed to assay several characteristic features of the RTT-like phenotype, including activity, gait, hind limb clasping, tremor, breathing, and general condition (Guy et al., 2007). Both models initially displayed a rapid onset of symptoms before remaining relatively stable from approximately 15 weeks. Strikingly, the trajectory of disease progression was extremely similar for both lines (Figure 4B), and we were unable to detect any statistically significant difference between the two groups at age 15 weeks—the last time point at which all animals were still alive (Figure S2C).

Finally, we investigated the molecular and histological phenotypes present in our mutant mice. To this end, we analyzed the expression levels of a panel of genes previously reported to be consistently downregulated (*Sst*, *Gabbr1*, and *Tspan17*) or upregulated (*Flrt3* and *Arhgap10*) across multiple brain regions and in several mouse models of RTT (Baker et al., 2013; Ben-Shachar et al., 2009; Mellén et al., 2012; Zhao et al., 2013). When compared to wild-type controls, both of our mouse lines showed subtle but statistically significant changes in the expression levels of these genes. However, for no tested genes were we able to detect any gene expression changes between our *Mecp2*^{G273X}-*EGFP* and *Mecp2*$^{G273X\Delta NLS}$-*EGFP* animals

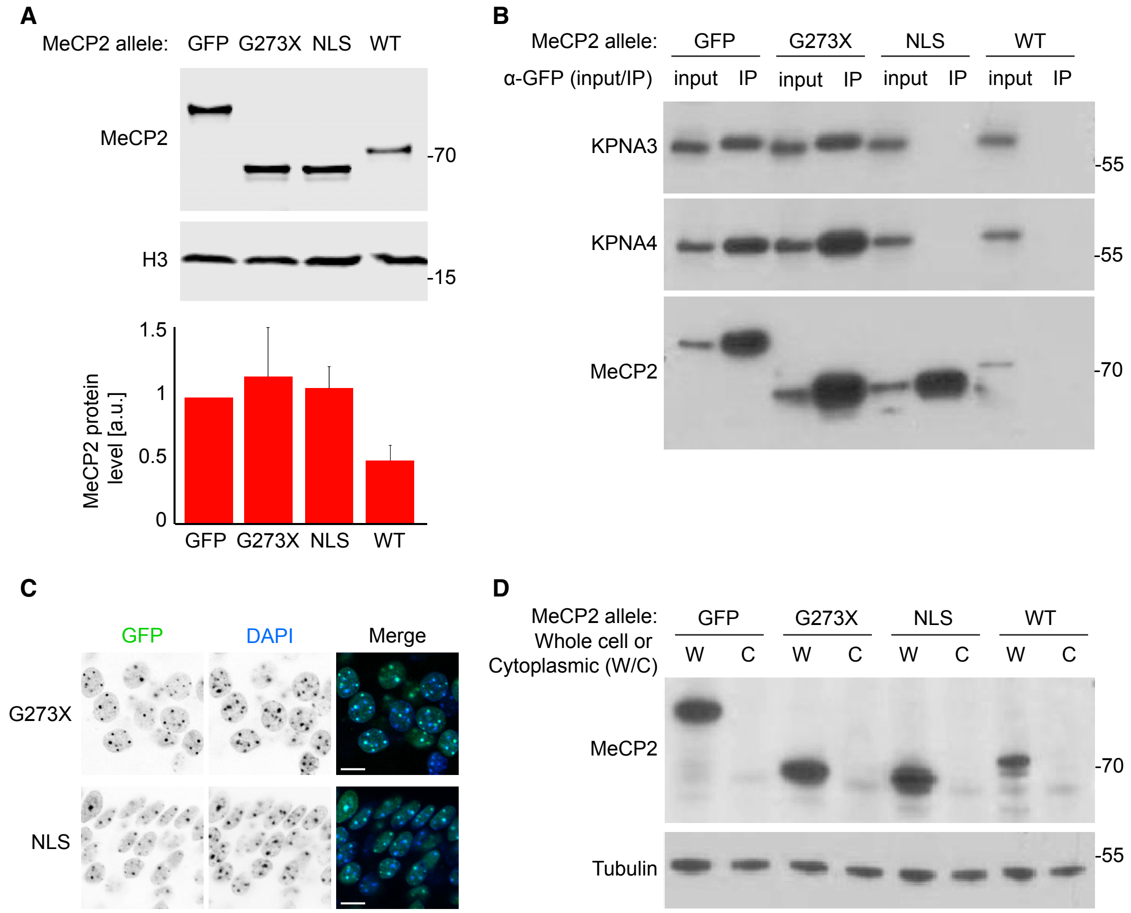

Figure 3. NLS Mutation Does Not Affect the Expression Level or Localization of MeCP2^{G273X}-EGFP in Mouse Brain

(A) Western blotting and quantification of MeCP2 levels in WT, *Mecp2-EGFP* (EGFP), *Mecp2^{G273X}-EGFP* (G273X), and *Mecp2$^{G273X\Delta NLS}$-EGFP* (NLS) whole-mouse brain extracts (n = 4). Loading control is anti-histone H3. Quantification is presented as means ± SD.

(B) IP of MeCP2-EGFP from extracts of brains from animals as in (A) using an antibody against EGFP. MeCP2 as well as bound Kpna3 and Kpna4 are detected by western blotting.

(C) Fluorescence microscopy of MeCP2-EGFP in CA1 layer of hippocampus in brain sections of *Mecp2^{G273X}-EGFP* (G273X), and *Mecp2$^{G273GX\Delta NLS}$-EGFP* (NLS) mice. The scale bars represent 10 μm.

(D) Western blot of MeCP2 levels in cytoplasmic (C) and whole-cell (W) fraction from WT, *Mecp2-EGFP* (EGFP), *Mecp2^{G273X}-EGFP* (G273X), and *Mecp2$^{G273GX\Delta NLS}$-EGFP* (NLS) mouse brain. Control cytoplasmic protein is gamma-tubulin.

(Figure 4C). To assess the histological phenotypes of our *Mecp2* mutant mouse lines, we examined the brain weights and cortical thicknesses of these animals. Mouse models of RTT have previously been shown to have smaller brains with reduced cortical thicknesses (Chen et al., 2001; Robinson et al., 2012). Consistent with these reports, we found that both the *Mecp2^{G273X}-EGFP* and *Mecp2$^{G273X\Delta NLS}$-EGFP* animals displayed reduced brain weights and reduced thickness of the primary motor cortex when compared to wild-type controls. However, we were unable to detect any changes in these features when we compared the two mutant lines with one another (Figures 4D and 4E).

In summary, by monitoring survival, weight, phenotypic severity score, gene expression profiles, and cortical thickness, we were not able to observe any additional deficits in *Mecp2$^{G273X\Delta NLS}$-EGFP* mice compared with *Mecp2^{G273X}-EGFP* controls. We conclude that importin binding does not substantially affect the course of the pathology in these models of RTT and that the NLS of MeCP2 does not therefore strongly contribute to the function of this protein.

DISCUSSION

MeCP2 is a nuclear factor whose ability to recruit the NCOR1/NCOR2 co-repressor complex to chromatin is essential for proper brain function. Given that MeCP2 resides in the nucleus, the absence of disease-causing mutations in its NLS may at first appear inconsistent with the fact that MeCP2 inactivation leads to Rett syndrome. We have resolved this paradox by uncovering a functional redundancy in the mechanism by which MeCP2 is targeted to the nuclear compartment. An alternative explanation for the lack of RTT-causing mutations in the NLS would be if spontaneous mutations inactivating the MeCP2 NLS do not

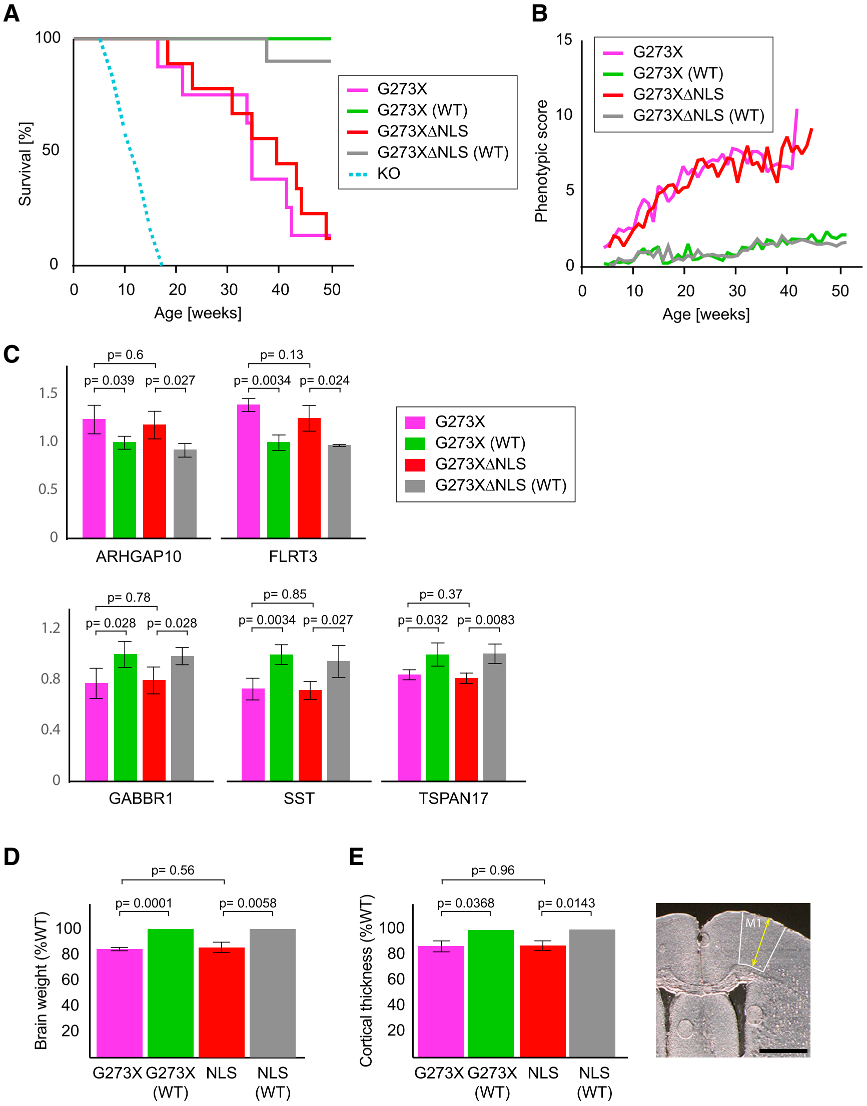

Figure 4. MeCP2^{G273X}-EGFP Mice Appear Unaffected by a Mutation Inactivating the MeCP2 NLS

(A) Survival curves for *Mecp2^{G273X}-EGFP* (n = 8) and *Mecp2$^{G273X\Delta NLS}$-EGFP* mice (n = 9), as well as wild-type controls for each group (n = 12 and n = 10, respectively). No significant difference between survival of *Mecp2^{G273X}-EGFP* and *Mecp2$^{G273X\Delta NLS}$-EGFP* mice was observed (p = 0.78; Kolmogorov-Smirnov test). An illustrative survival curve for *Mecp2*-null mice (based on data from previous studies) is shown as a broken line.

(B) Average phenotypic severity score of surviving animals plotted against age for each of the groups. Data for each group are plotted up to the last point, where there are at least three surviving animals.

(C) Expression levels relative to *Gapdh* of genes that are upregulated (*Flrt3* and *Arhgap10*) or downregulated (*Sst*, *Tspan17*, and *Gabbr1*) in brains of *Mecp2* mutant mice as assessed by qPCR. Data are presented as means ± SD.

(D) Brain weights for *Mecp2^{G273X}-EGFP* and *Mecp2$^{G273X\Delta NLS}$-EGFP* mice (n = 4 for each group) as well as wild-type controls (n = 5 and n = 3). Indicated statistical significance was calculated using the t test. Data are presented as means ± SD.

(E) Cortical thickness plotted for *Mecp2^{G273X}-EGFP* and *Mecp2$^{G273X\Delta NLS}$-EGFP* mice (n = 4 each) and wild-type control animals (n = 5 and n = 3). Data are presented as means ± SD. Indicated statistical significance was calculated using the t test. The image depicts the area of the primary motor cortex (M1) measured. The scale bar represents 1,000 μm.

See also Figure S2.

occur in the human population. However, this appears unlikely, given the demonstration that a single amino acid substitution in the NLS can abolish its interaction with the nuclear import machinery (Baker et al., 2015).

The NLS of MeCP2 was originally defined by expressing fragments of MeCP2 as beta-galactosidase fusion proteins and assaying their localization in mouse fibroblasts (Nan et al., 1996). In this system, the NLS was necessary for MeCP2 to be partitioned in the nucleus. At first sight, these data contradict our finding that the MeCP2 NLS is dispensable for its nuclear localization. A likely explanation for the discrepancy is that beta-galactosidase, a large 464-kDa homo-tetramer, fused to MeCP2 might obligatorily require active transport through the nuclear pore complex. According to this hypothesis, smaller MeCP2 fusions with EGFP would be capable of NLS-independent entry into the nucleus, where they are then retained by their affinity for DNA. Although we cannot formally exclude the possibility that the DNA binding domains of MeCP2 interact with the nuclear import machinery to mediate active transport of this protein into the nucleus, we consider this less likely. First, we find no evidence for these domains interacting with the nuclear import machinery (Figure 2C). Second, two independent DNA binding domains, the MBD and AT-hook 1, act synergistically to direct nuclear localization. These two domains are structurally unrelated, but each has an affinity for a subset of nucleotide sequence motifs that is frequent in the genome.

Given that the MeCP2 NLS appears not to be required for localization of MeCP2 in the nucleus, a question arises as to the function of its interaction with KPNA3 and KPNA4. The NLS is a highly conserved region of MeCP2, and so its inactivation is likely to be detrimental, even if only on evolutionary timescales. One possibility is that importin binding to MeCP2 reflects a non-canonical role of these proteins. Importins have been proposed to function as chaperones for certain factors (Jäkel et al., 2002), and KPNA3/KPNA4 binding to MeCP2 could also reflect such a role. Alternatively, the selection pressure on the MeCP2 NLS may arise due to an extremely subtle effect on the nuclear targeting of this protein. For example, the kinetics of MeCP2 nuclear import might be perturbed or an extremely low

concentration of MeCP2 in the cytoplasm of large neurons might reduce the amount of active protein available in the nucleus. Whatever the role of MeCP2 binding to the nuclear import machinery, our work has highlighted the importance of using genetics to avoid misattributing disease relevance to evolutionarily conserved biochemical interactions.

This study has uncovered a surprising redundancy between the DNA binding domains and the NLS of MeCP2 in the nuclear localization of this factor. Given the paucity of NLS-inactivating mutations implicated in other genetic disorders (McLane and Corbett, 2009), an interesting line of future work will be to determine whether chromatin binding also contributes to nuclear sequestration of other factors. As well as providing insights into the mechanism of nuclear targeting of MeCP2, the present study should also be of interest to researchers seeking treatments for Rett syndrome. Currently, the gene therapy vectors showing the most promise in targeting the CNS have a limited capacity, leading to efforts to miniaturize MeCP2 expression constructs containing only the required functional domains (Tillotson et al., 2017). By better delineating the disease-relevant functional domains of MeCP2, our work has both improved molecular understanding of this protein and also strengthened the groundwork for one of the more promising strategies for treating Rett syndrome.

EXPERIMENTAL PROCEDURES

Protein Interactions

Co-immunoprecipitation and *in vitro* binding assays were performed essentially as described previously (Lyst et al., 2013), except that buffers were supplemented with 300 mM rather than 150 mM sodium chloride where indicated. GST fusion proteins were produced as described elsewhere (Lyst et al., 2016).

Cell Imaging

NIH 3T3 cells were maintained, transfected, harvested, fixed, and imaged as described previously (Lyst et al., 2013). LUHMES cells were maintained and transduced with lentiviruses as described previously (Shah et al., 2016). Images were quantified using the ImageJ software package with the investigator blind to the identities of the transfected MeCP2 constructs. ΔMBD and ΔMBDΔAT-hook 1 constructs lacked amino acids 111–168 and 111–191.

Subcellular Fractionation

For preparation of whole brain and cytoplasmic brain extracts, fresh material (not frozen) was dounce homogenized in NE1 buffer (20 mM HEPES [pH 7.5], 10 mM NaCl, 1 mM MgCl2, and 0.1% Triton X-100). This homogenate was taken as whole-brain extract, and the supernatant following centrifugation for 5 min at 500 g was taken as cytoplasmic extract.

Animals

All animal experiments were carried out by staff licensed by the UK Home Office in accordance with the Animal and Scientific Procedures Act 1986. Targeting vectors for generating mutant mice were based on one described elsewhere (Lyst et al., 2013). For the NLS mutation, four consecutive amino acids (RKRK) comprising the N-terminal basic cluster of this bipartite signal were mutated to alanine. Targeting vector was co-transfected into 129/Ola E14 TG2a mouse embryonic stem cells (ESCs) with Cas9 (nicking) and appropriate guide RNA expression plasmids (Ran et al., 2013). Mice were generated from these cells by standard procedures before backcrossing onto the C57BL/6 background for at least two generations. Scoring of phenotypes was performed blind to genotypes as described elsewhere (Guy et al., 2007), with wild-type and mutant male mice being weighed and monitored weekly from 4 weeks of age for one year. For biochemical analysis (co-immunoprecipitation, subcellular fractionation, and western blotting), brains were taken from male mice aged 8–18 weeks.

Histology

Brains from wild-type and mutant male littermates aged 10–18 weeks were dissected, removing the olfactory bulb and trimming to remove any spinal cord. Brains were weighed and immersed in fixative (3.7% paraformaldehyde in PBS). After 2 hr, brains were transferred to fresh fixative and stored overnight at 4°C. Brains were transferred to 30% sucrose solution in PBS and stored at 4°C for 48 hr, replacing the solution after 24 hr. Brains were then frozen in isopentane, chilled on dry ice, and stored at −80°C. A Leica CM1900 cryostat set to −26°C was used to cut 30-μm coronal sections, which were mounted on SuperFrost Plus slides and dried. Sections were photographed using an Olympus SZX10 microscope at 2.6× magnification. The location of each section was determined with reference to "The Mouse Brain in Stereotaxic Coordinates" (Franklin and Paxinos, 2008). The primary motor cortex was defined using the atlas and the cortical thickness measured near to the center of the region. Between 22 and 33 sections were measured for each mouse, between 0.98 and −0.46 mm from Bregma (Franklin and Paxinos, 2008).

Gene Expression Analysis

Total RNA was purified from whole brains of male mice aged 7–14 weeks using TRI Reagent (Sigma; T9424). The QuantiTect Reverse Transcription Kit (QIAGEN) was used for cDNA synthesis, and gene expression was quantified using the SensiMix SYBR & Fluorescein kit (Bioline) on a LightCycler 480.

Statistical Methods

For quantification of the nuclear to cytoplasmic ratio of EGFP-MeCP2 mutants in NIH 3T3 cells, statistical significance was calculated using the Wilcoxon test. For qPCR data as well as analysis of brain weights and cortical thicknesses, p values were calculated using the Student's t test. The Kolmogorov-Smirnov test was used to calculate p values for comparisons of body weights and phenotypic scores.

Antibodies

Antibodies used were as follows: Histone H3 (Abcam; ab1791); gamma-tubulin (Sigma; T5326); MeCP2 (Sigma; M6818 and M7443); KPNA3 (Abcam; ab6038); KPNA4 (Bethyl Laboratories; A301-627A); EGFP (CRUK and Chromotek gta-10); and FLAG (Sigma; F3165).

SUPPLEMENTAL INFORMATION

Supplemental Information includes two figures and can be found with this article online at https://doi.org/10.1016/j.celrep.2018.07.099.

ACKNOWLEDGMENTS

We thank members of the Bird lab for helpful discussions and Weronika Borek for critical reading of the manuscript. This work was supported by Wellcome (investigator award 107930 to A.B., four year PhD studentship 083215 to R.E., and core funding 203149) and the Rett Syndrome Research Trust.

AUTHOR CONTRIBUTIONS

M.J.L. performed biochemical, cell imaging, and gene expression analysis. R.E. mapped KPNA3/4 binding site to the NLS of MeCP2. C.M. generated embryonic stem cell lines. J.S. generated mouse lines. J.G. performed histology. M.V.K. and D.D.S. performed phenotypic analysis of mice. M.J.L. and A.B. conceived the experiments and wrote the paper.

DECLARATION OF INTERESTS

The authors declare no competing interests.

Received: December 13, 2017
Revised: June 22, 2018
Accepted: July 26, 2018
Published: August 28, 2018

REFERENCES

Amir, R.E., Van den Veyver, I.B., Wan, M., Tran, C.Q., Francke, U., and Zoghbi, H.Y. (1999). Rett syndrome is caused by mutations in X-linked MECP2, encoding methyl-CpG-binding protein 2. Nat. Genet. *23*, 185–188.

Baker, S.A., Chen, L., Wilkins, A.D., Yu, P., Lichtarge, O., and Zoghbi, H.Y. (2013). An AT-hook domain in MeCP2 determines the clinical course of Rett syndrome and related disorders. Cell *152*, 984–996.

Baker, S.A., Lombardi, L.M., and Zoghbi, H.Y. (2015). Karyopherin α 3 and karyopherin α 4 proteins mediate the nuclear import of methyl-CpG binding protein 2. J. Biol. Chem. *290*, 22485–22493.

Baubec, T., Ivánek, R., Lienert, F., and Schübeler, D. (2013). Methylation-dependent and -independent genomic targeting principles of the MBD protein family. Cell *153*, 480–492.

Ben-Shachar, S., Chahrour, M., Thaller, C., Shaw, C.A., and Zoghbi, H.Y. (2009). Mouse models of MeCP2 disorders share gene expression changes in the cerebellum and hypothalamus. Hum. Mol. Genet. *18*, 2431–2442.

Brown, K., Selfridge, J., Lagger, S., Connelly, J., De Sousa, D., Kerr, A., Webb, S., Guy, J., Merusi, C., Koerner, M.V., and Bird, A. (2016). The molecular basis of variable phenotypic severity among common missense mutations causing Rett syndrome. Hum. Mol. Genet. *25*, 558–570.

Chahrour, M., Jung, S.Y., Shaw, C., Zhou, X., Wong, S.T.C., Qin, J., and Zoghbi, H.Y. (2008). MeCP2, a key contributor to neurological disease, activates and represses transcription. Science *320*, 1224–1229.

Chen, R.Z., Akbarian, S., Tudor, M., and Jaenisch, R. (2001). Deficiency of methyl-CpG binding protein-2 in CNS neurons results in a Rett-like phenotype in mice. Nat. Genet. *27*, 327–331.

Cheng, T.L., Wang, Z., Liao, Q., Zhu, Y., Zhou, W.H., Xu, W., and Qiu, Z. (2014). MeCP2 suppresses nuclear microRNA processing and dendritic growth by regulating the DGCR8/Drosha complex. Dev. Cell *28*, 547–560.

Christodoulou, J., Grimm, A., Maher, T., and Bennetts, B. (2003). RettBASE: the IRSA MECP2 variation database-a new mutation database in evolution. Hum. Mutat. *21*, 466–472.

Franklin, K.B.J., and Paxinos, G. (2008). The Mouse Brain in Stereotaxic Coordinates, Third Edition (Academic).

Gabel, H.W., Kinde, B., Stroud, H., Gilbert, C.S., Harmin, D.A., Kastan, N.R., Hemberg, M., Ebert, D.H., and Greenberg, M.E. (2015). Disruption of DNA-methylation-dependent long gene repression in Rett syndrome. Nature *522*, 89–93.

Goffin, D., Allen, M., Zhang, L., Amorim, M., Wang, I.-T.J., Reyes, A.-R.S., Mercado-Berton, A., Ong, C., Cohen, S., Hu, L., et al. (2011). Rett syndrome mutation MeCP2 T158A disrupts DNA binding, protein stability and ERP responses. Nat. Neurosci. *15*, 274–283.

Guy, J., Hendrich, B., Holmes, M., Martin, J.E., and Bird, A. (2001). A mouse Mecp2-null mutation causes neurological symptoms that mimic Rett syndrome. Nat. Genet. *27*, 322–326.

Guy, J., Gan, J., Selfridge, J., Cobb, S., and Bird, A. (2007). Reversal of neurological defects in a mouse model of Rett syndrome. Science *315*, 1143–1147.

Jäkel, S., Mingot, J.-M., Schwarzmaier, P., Hartmann, E., and Görlich, D. (2002). Importins fulfil a dual function as nuclear import receptors and cytoplasmic chaperones for exposed basic domains. EMBO J. *21*, 377–386.

Kinde, B., Wu, D.Y., Greenberg, M.E., and Gabel, H.W. (2016). DNA methylation in the gene body influences MeCP2-mediated gene repression. Proc. Natl. Acad. Sci. USA *113*, 15114–15119.

Kruusvee, V., Lyst, M.J., Taylor, C., Tarnauskaitė, Ž., Bird, A.P., and Cook, A.G. (2017). Structure of the MeCP2-TBLR1 complex reveals a molecular basis for Rett syndrome and related disorders. Proc. Natl. Acad. Sci. USA *114*, E3243–E3250.

Kumar, A., Kamboj, S., Malone, B.M., Kudo, S., Twiss, J.L., Czymmek, K.J., LaSalle, J.M., and Schanen, N.C. (2008). Analysis of protein domains and Rett syndrome mutations indicate that multiple regions influence chromatin-binding dynamics of the chromatin-associated protein MECP2 in vivo. J. Cell Sci. *121*, 1128–1137.

Lagger, S., Connelly, J.C., Schweikert, G., Webb, S., Selfridge, J., Ramsahoye, B.H., Yu, M., He, C., Sanguinetti, G., Sowers, L.C., et al. (2017). MeCP2 recognizes cytosine methylated tri-nucleotide and di-nucleotide sequences to tune transcription in the mammalian brain. PLoS Genet. *13*, e1006793.

Lewis, J.D., Meehan, R.R., Henzel, W.J., Maurer-Fogy, I., Jeppesen, P., Klein, F., and Bird, A. (1992). Purification, sequence, and cellular localization of a novel chromosomal protein that binds to methylated DNA. Cell *69*, 905–914.

Li, Y., Wang, H., Muffat, J., Cheng, A.W., Orlando, D.A., Lovén, J., Kwok, S.-M., Feldman, D.A., Bateup, H.S., Gao, Q., et al. (2013). Global transcriptional and translational repression in human-embryonic-stem-cell-derived Rett syndrome neurons. Cell Stem Cell *13*, 446–458.

Lyst, M.J., and Bird, A. (2015). Rett syndrome: a complex disorder with simple roots. Nat. Rev. Genet. *16*, 261–275.

Lyst, M.J., Ekiert, R., Ebert, D.H., Merusi, C., Nowak, J., Selfridge, J., Guy, J., Kastan, N.R., Robinson, N.D., de Lima Alves, F., et al. (2013). Rett syndrome mutations abolish the interaction of MeCP2 with the NCoR/SMRT co-repressor. Nat. Neurosci. *16*, 898–902.

Lyst, M.J., Connelly, J., Merusi, C., and Bird, A. (2016). Sequence-specific DNA binding by AT-hook motifs in MeCP2. FEBS Lett. *590*, 2927–2933.

McLane, L.M., and Corbett, A.H. (2009). Nuclear localization signals and human disease. IUBMB Life *61*, 697–706.

Mellén, M., Ayata, P., Dewell, S., Kriaucionis, S., and Heintz, N. (2012). MeCP2 binds to 5hmC enriched within active genes and accessible chromatin in the nervous system. Cell *151*, 1417–1430.

Nan, X., Meehan, R.R., and Bird, A. (1993). Dissection of the methyl-CpG binding domain from the chromosomal protein MeCP2. Nucleic Acids Res. *21*, 4886–4892.

Nan, X., Tate, P., Li, E., and Bird, A. (1996). DNA methylation specifies chromosomal localization of MeCP2. Mol. Cell. Biol. *16*, 414–421.

Ran, F.A., Hsu, P.D., Lin, C.Y., Gootenberg, J.S., Konermann, S., Trevino, A.E., Scott, D.A., Inoue, A., Matoba, S., Zhang, Y., and Zhang, F. (2013). Double nicking by RNA-guided CRISPR Cas9 for enhanced genome editing specificity. Cell *154*, 1380–1389.

Robinson, L., Guy, J., McKay, L., Brockett, E., Spike, R.C., Selfridge, J., De Sousa, D., Merusi, C., Riedel, G., Bird, A., and Cobb, S.R. (2012). Morphological and functional reversal of phenotypes in a mouse model of Rett syndrome. Brain *135*, 2699–2710.

Schmiedeberg, L., Skene, P., Deaton, A., and Bird, A. (2009). A temporal threshold for formaldehyde crosslinking and fixation. PLoS ONE *4*, e4636.

Scholz, D., Pöltl, D., Genewsky, A., Weng, M., Waldmann, T., Schildknecht, S., and Leist, M. (2011). Rapid, complete and large-scale generation of post-mitotic neurons from the human LUHMES cell line. J. Neurochem. *119*, 957–971.

Shah, R.R., Cholewa-Waclaw, J., Davies, F.C.J., Paton, K.M., Chaligne, R., Heard, E., Abbott, C.M., and Bird, A.P. (2016). Efficient and versatile CRISPR engineering of human neurons in culture to model neurological disorders. Wellcome Open Res. *1*, 13.

Sorokin, A.V., Kim, E.R., and Ovchinnikov, L.P. (2007). Nucleocytoplasmic transport of proteins. Biochemistry (Mosc.) *72*, 1439–1457.

Tillotson, R., Selfridge, J., Koerner, M.V., Gadalla, K.K.E., Guy, J., De Sousa, D., Hector, R.D., Cobb, S.R., and Bird, A. (2017). Radically truncated MeCP2 rescues Rett syndrome-like neurological defects. Nature *550*, 398–401.

Young, J.I., Hong, E.P., Castle, J.C., Crespo-Barreto, J., Bowman, A.B., Rose, M.F., Kang, D., Richman, R., Johnson, J.M., Berget, S., and Zoghbi, H.Y. (2005). Regulation of RNA splicing by the methylation-dependent transcriptional repressor methyl-CpG binding protein 2. Proc. Natl. Acad. Sci. USA *102*, 17551–17558.

Zhao, Y.-T., Goffin, D., Johnson, B.S., and Zhou, Z. (2013). Loss of MeCP2 function is associated with distinct gene expression changes in the striatum. Neurobiol. Dis. *59*, 257–266.

Cell Reports, Volume *24*

Supplemental Information

Affinity for DNA Contributes to NLS Independent Nuclear Localization of MeCP2

Matthew J. Lyst, Robert Ekiert, Jacky Guy, Jim Selfridge, Martha V. Koerner, Cara Merusi, Dina De Sousa, and Adrian Bird

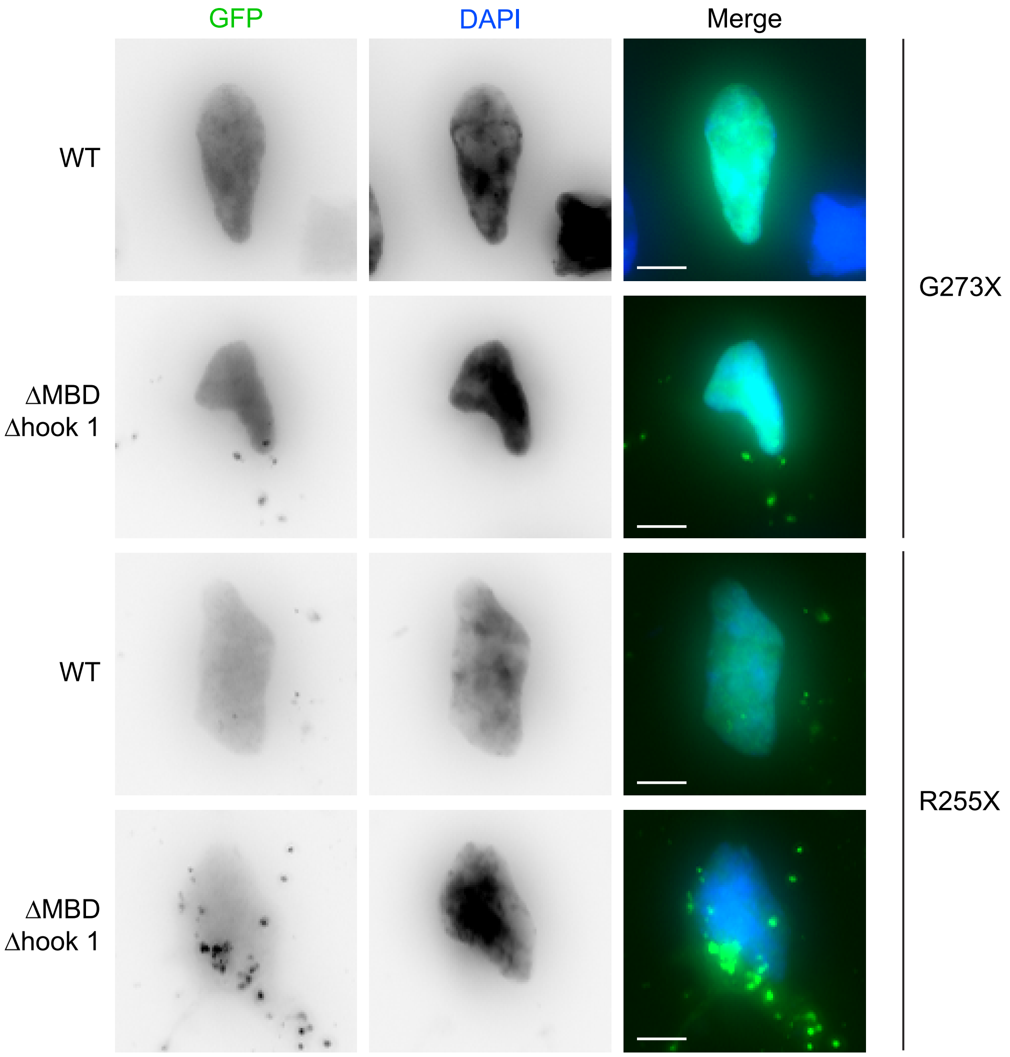

Figure S1. Redundancy between DNA binding and the NLS for nuclear localization of MeCP2 expressed in the human neuronal LUHMES cell line. Related to Figure 2.

Lentivirally expressed EGFP-MeCP2 with an intact NLS (G273X truncation) is nuclear and co-localizes with DAPI stain regardless of whether DNA binding is wild-type (WT) (top panels) or disturbed (ΔMBDΔhook 1) (second panels). When the NLS is removed (R255X truncation) EGFP-MeCP2 remains nuclear when the DNA binding domains are intact (WT) (third panels), but becomes largely cytoplasmic when the DNA binding domains are mutated (ΔMBDΔhook 1) (bottom panels). The scale bar represents 5 μm.

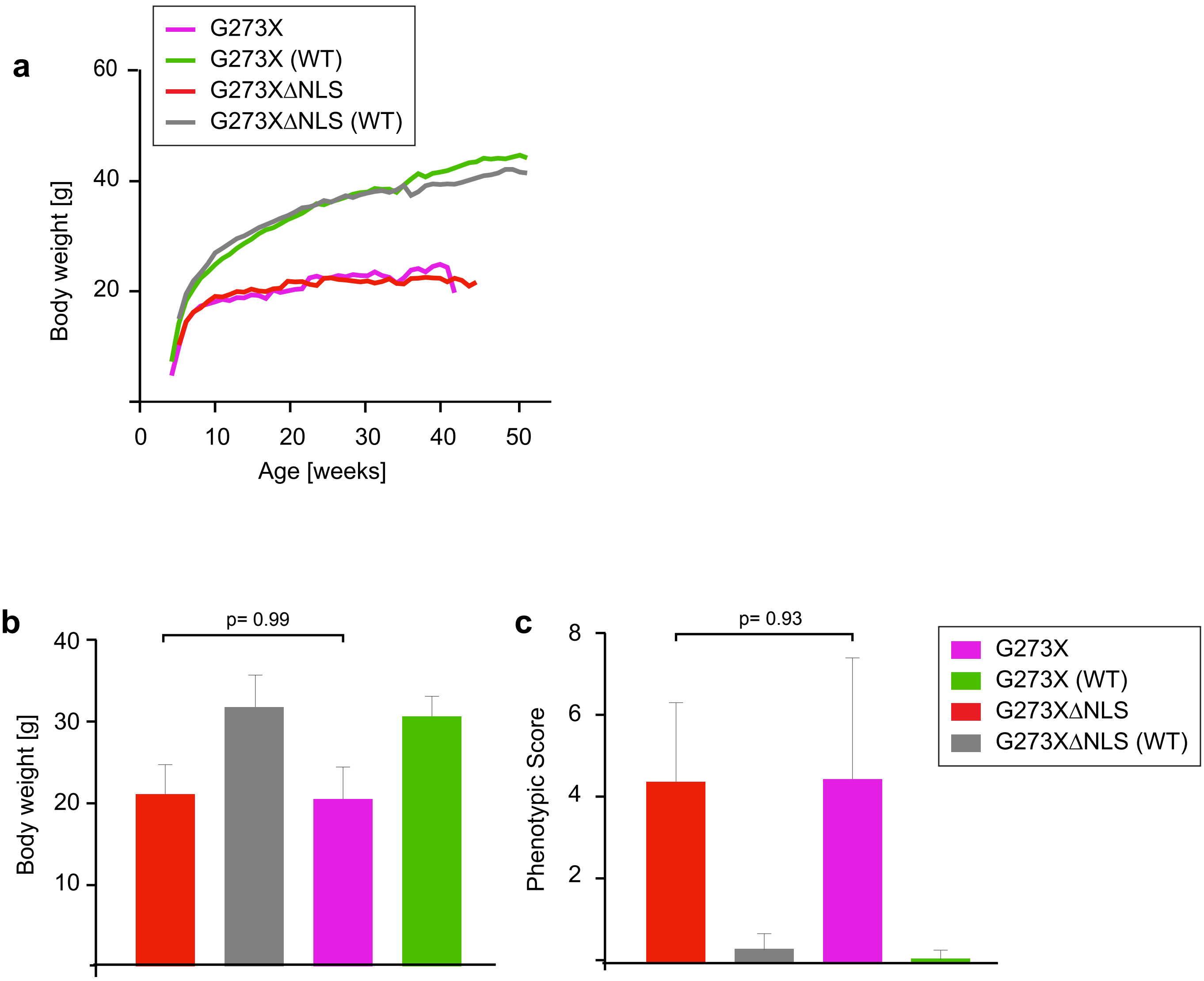

Figure S2. Body weights and phenotypic scores of MeCP2^{G273X}-EGFP and MeCP2$^{G273X\Delta NLS}$-EGFP mice. Related to Figure 4.

(a) Average body weight of surviving animals is plotted against age for each group. Data for each group plotted up to the last point when there were at least three surviving animals. **(b)** Body weights of each group of animals at 15-weeks – the last time point to which all mice survived. Data are presented as means ± standard deviation. No significant difference was observed between MeCP2^{G273X}-EGFP and MeCP2$^{G273X\Delta NLS}$-EGFP mice ($p=0.99$; Kolmogorov-Smirnov test). **(c)** Phenotypic scores of each group of animals at 15-weeks – the last time point to which all of the mice survived. Data are presented as means ± standard deviation. No significant difference was observed between MeCP2^{G273X}-EGFP and MeCP2$^{G273X\Delta NLS}$-EGFP mice ($p=0.93$; Kolmogorov-Smirnov test).
